# Factors influencing antimicrobial stewardship implementation in healthcare facilities in four countries of Southeast Asia: a mixed-methods study (2022–23)

**DOI:** 10.1016/j.lansea.2026.100837

**Published:** 2026-08-06

**Authors:** Ralalicia Limato, Sulochana Manandhar, Direk Limmathurotsakul, Anucha Apisarnthanarak, Raph L. Hamers, Erni J. Nelwan, Abhilasha Karkey, Twisha S. Patel, Fernanda C. Lessa, Elizabeth Dodds Ashley, Deverick J. Anderson, Hai Yen Duong, Van Giang Tran, H Rogier van Doorn, Huong T.L. Vu, G-ASET Development and Implementation Group in Asia, Raph L. Hamers, Ralalicia Limato, Elza Samantha Elmira, Fariz R. Iswoyo, Ivan Damara, Korrie Salsabila, Marita Joesoef, Muhammad RH. Lubis, Talitha V. Salsabella, Tamara Tango, Taris Radifan, Enty Tjoa, Erni Juwita Nelwan, Robert Sinto, Sharifah Shakinah, Abhilasha Karkey, Sulochana Manandhar, Sushmita Shrestha, Alisha Pradhan, Binod Bijukchhe, Prashant Tripathi, Basudha Khanal, Sanjib Kumar Sharma, Narayan Raj Bhattarai, Ratna Baral, Shyam Prasad Kafle, Piyush Dahal, Dipendra Kumar Raushan, Gyan Kayastha, Maya Bade, Direk Limmathurotsakul, Pornpan Sunthornsut, Anucha Apisarnthanarak, Amornrat Vijitleela, Nuntiya Somjetanakul, Anond Kulthanmanusorn, Lantharita Charoenpong, Suwimon Khusuwan, Suchada Ruenglertphong, Phiradee Chanthirawong, Patcharin Sumanapunyawong, Sureeruk Achalapong, Suwatthiya Kitsaran, Patcharee Karnjanawat, Korawan Pudpong, Ramphai Sukkasem, Somsamai Boonsong, Huong TL. Vu, H Rogier van Doorn, Hai Yen Duong, Thi Thu Hien Phan, Thi Cam Tu Nguyen, Anh Quan Truong, Hai Yen Nguyen, Thanh Ha Nguyen, Ngoc Thach Pham, Van Cap Nguyen, Van Giang Tran, Nguyen Minh Hoa Le, Thi Thuy Van Pham, Thi Thu Huong Nguyen, Thi Chi Dinh, Ba Hien Pham, Thi Phuong Thuy Nguyen, Thu Hang Nguyen, Anh Cuong Tran, Thi Ngoc Diep Nguyen, Thi Thuy Nguyen, Xuan Nam Trinh, Ngoc Hoa Nguyen, Anh Dao Tran, Thi Lan Huong Hoang, Van Tuan Mai, Minh Duc Chau, Thi Hoang Dung Em Vo, Hung Thai Cao, Christian Shaw, Deverick J. Anderson, Elizabeth Dodds Ashley, Fernanda C. Lessa, Twisha S. Patel

**Affiliations:** a Oxford University Clinical Research Unit Indonesia, Faculty of Medicine Universitas Indonesia, Jakarta, Indonesia; b Centre for Tropical Medicine and Global Health, Nuffield Department of Medicine, University of Oxford, Oxford, UK; c Oxford University Clinical Research Unit Nepal, Kathmandu, Nepal; d Mahidol Oxford Tropical Medicine Research Unit, Faculty of Tropical Medicine, Mahidol University, Bangkok, Thailand; e Faculty of Medicine, Thammasat University Hospital, Pratumthani, Thailand; f Faculty of Medicine, Universitas Indonesia, Jakarta, Indonesia; g Division of Infectious Diseases, Department of Internal Medicine, Cipto Mangunkusumo National General Hospital, Jakarta, Indonesia; h Division of Healthcare Quality Promotion, U.S. Centers for Disease Control and Prevention, United States; i Duke Antimicrobial Stewardship Outreach Network, Duke Center for Antimicrobial Stewardship and Infection Prevention, Duke University, Durham, NC, 27710, United States; j Oxford University Clinical Research Unit Viet Nam, Thon Bau, Thien Loc, Hanoi, Viet Nam; k Hanoi Medical University, No. 1 Ton That Tung, Kim Lien, Hanoi, Viet Nam; l National Hospital for Tropical Diseases, Thon Bau, Thien Loc, Hanoi, Viet Nam

**Keywords:** Antimicrobial stewardship, Antimicrobial resistance, Implementation, Healthcare facilities, Low- and middle-income countries, South and Southeast Asia

## Abstract

**Background:**

Antimicrobial stewardship (AMS) implementation is highly context dependent. This mixed-methods study aimed to assess contextual factors associated with AMS implementation at national, facility, and healthcare-provider levels in four countries.

**Methods:**

We used a sequential mixed-methods approach, with a combination of quantitative surveys, qualitative interviews and focus group discussions (FGDs) using the World Health Organisation's National AMS assessment tools at national, healthcare-facility, and provider levels in Indonesia, Nepal, Thailand, and Viet Nam between 2022 and 2023. Facility-level implementation was evaluated using the U.S. Centers for Disease Control and Prevention's Global Antibiotic Stewardship Evaluation Tool (G-ASET) in 16 hospitals across these countries. We included stakeholders and healthcare personnel involved in AMS implementation at the national and hospital levels.

**Findings:**

All four countries had national AMS policies, and two incorporated financing mechanisms into national action plans. Main areas for further improvement were the development of stewardship guidelines for primary care and community settings; stronger linkages across related sectors and programmes; expanded professional training; strengthened regulatory enforcement; systematic performance tracking; and better data use for feedback and decision-making. Among the 16 hospitals, the mean G-ASET score was 241/335 (72%), highest in tracking, monitoring and reporting [56/75 (75%)] and lowest in training and education [17/25 (66%)], and the scores varied by hospital type and size. Although all hospitals had initiated AMS activities, implementation was often partial, with gaps in data-informed treatment guidelines, staff training, and resources.

**Interpretation:**

National guidelines on treatment and stewardship initiatives are crucial and trainings for healthcare personnel need to be strengthened for facility-level AMS implementation. Enhancing inter-programme coordination and support for small-size hospitals and primary care settings in Asia will be essential to advance AMS implementation.

**Funding:**

Supported by the U.S. Centers for Disease Control and Prevention.

**Research in context**

**Evidence before this study**

We searched PubMed for papers published up to 8 July 2026, using keywords related to antimicrobial stewardship (AMS) implementation, barriers and facilitators, hospital settings, reviews, and low- and middle-income countries (LMICs) or resource-limited settings. Our search identified 25 records, comprising reviews and implementation-focused papers on hospital-based AMS in LMICs. Recurrent barriers to AMS implementation included limited leadership and governance, inadequate training, weak microbiology and surveillance capacity, poor data systems, staffing constraints, prescriber attitudes, antibiotic shortages, and patient demand for antibiotics. Facilitators included national guidance, local guidelines, audit and feedback, multidisciplinary stewardship teams, and stronger access to laboratory data. However, implementation challenges in resource-constrained settings are not well characterised. Our previous scoping review of global AMS guidance documents (2005–2021) highlighted that existing guidance did not adequately address AMS implementation barriers in LMICs. Subsequently, the Global Antibiotic Stewardship Evaluation Tool (G-ASET) for inpatient healthcare facilities was developed through expert consensus to assess AMS implementation at the hospital level.

**Added value of this study**

This mixed-methods study contributes to the evidence on the implementation of AMS programmes in LMICs by examining facilitators and barriers at national, facility, and healthcare-provider levels utilising tools such as the WHO national assessment tool, G-ASET, and knowledge, attitudes, and practices (KAP) survey. Although all four countries had national AMS policies, local implementation was constrained by gaps in inter-programme linkages, training, microbiology data use, and resources. At the hospital level, leadership support was important, but implementation remained partial in many settings, particularly in relation to updated treatment guidelines, standard operating procedures, pre-authorisation policies, and selective or cascading susceptibility reporting. Focus group discussions further highlighted the importance of local microbiology data, timely diagnostic and reporting services, antibiotic availability, multidisciplinary teamwork, and pressure to prescribe in response to patient demand and fear of treatment failure.

**Implications of all the available evidence**

The available evidence supports a comprehensive, implementation-oriented approach to AMS that combines national guidance with investment in workforce training, laboratory capacity, and data systems. It also reinforces the value of context-specific assessment tools, such as the U.S. CDC's G-ASET, for identifying actionable gaps in hospital AMS implementation and informing policy and resource allocation in LMICs.

## Introduction

Antimicrobial resistance (AMR) is a global public health threat, with a disproportionate burden in low- and middle-income countries (LMICs), including the South and Southeast Asia region, where antibiotic misuse is common and access to effective antimicrobial therapy remains limited.^1^, ^2^, ^3^ Antimicrobial stewardship (AMS) initiatives are therefore essential to optimise antibiotic use, improve patient outcomes, and prevent the emergence of resistance. Efficient hospital-based AMS programmes have resulted in substantial clinical and financial gains, such as reductions in unnecessary antibiotic use, lowering of healthcare-related costs, decreased incidence of drug resistance and healthcare-associated infections, and shorter hospital stays.^2^^,^^4^, ^5^, ^6^

AMS implementation requires adequate infrastructure, hospital leadership support, dedicated healthcare personnel with AMS-specialised training, and context-specific strategies.^7^ However, as shown in our recent scoping review, global AMS guidelines have been primarily designed based on experience from high-income settings.^3^ Many healthcare facilities in low- and middle-income countries (LMICs) are struggling to meet these requirements and therefore encounter more challenges in implementing AMS strategies effectively.^8^^,^^9^ In many LMICs, gaps in national-level policies, surveillance, funding, training, laboratory capacity, and antibiotic regulation can hinder AMS implementation, and these weaknesses often cascade to hospitals as poor leadership, inadequate training, limited microbiology services, suboptimal infection control, delayed antibiotic access, and conflicting economic incentives.^9^, ^10^, ^11^, ^12^, ^13^, ^14^ These issues collectively impact prescribing practices at the individual level,^9^ where prescribers face pressures such as diagnostic uncertainty, fear of adverse outcomes, and patient demands, leading to unnecessary antibiotic prescribing.

Considering the influence of multiple factors on antimicrobial resistance, this study aimed to assess the contextual factors associated with AMS implementation in four LMICs in South and Southeast Asia at three levels (national, healthcare facility, and individual healthcare provider), and to identify priority targets for improved AMS programme implementation in resource-limited settings.

## Methods

This study was part of a U.S. Centers for Disease Control and Prevention (U.S. CDC)-led global initiative that aimed to develop a context-appropriate global assessment tool–The Global Antibiotic Stewardship Evaluation Tool (G-ASET) for inpatient healthcare facilities–and assess AMS implementation in more than 80 hospitals across 12 countries in Asia, Africa and Latin America.^15^ In this study we included 16 hospitals in four countries: two upper-middle-income (Indonesia and Thailand) and two lower middle-income countries (Nepal and Viet Nam), selected based on pre-existing collaborative partnerships to represent South and Southeast Asia region. Two experts from the Duke Antimicrobial Stewardship Outreach Network (DASON) provided technical guidance and support for implementation.

We used a sequential mixed-methods approach, with a combination of quantitative surveys, qualitative interviews and focus group discussions (FGDs), aimed at performing a comprehensive assessment of AMS implementation and the associated factors at national, healthcare-facility, and provider levels (Figure S1-Supplementary File). The integration and sequence of data collection methods was designed to capture all relevant elements and provide an in-depth understanding of the critical nuances affecting AMS implementation in the local contexts. This paper follows established reporting standards for mixed-methods and qualitative studies: Good Reporting of A Mixed Methods Study (GRAMMS) checklist^16^ and the Consolidated criteria for reporting qualitative studies (COREQ).^17^

At the national level, we invited stakeholders involved in AMR-related policy development, including national action plans, to participate in the completion of a national-level assessment form and in-depth interviews. At the hospital level, we invited the AMS teams, or where teams are not formally established, relevant staff involved in AMS activities, of 16 hospitals (11 public and five private) in four countries to complete the G-ASET and semi-structured interviews. The number of hospitals included in the study were, five in Indonesia, three in Nepal, two in Thailand, and six in Viet Nam, selected based on our preexisting research collaboration networks. We aimed to include hospitals with variation in type (public and private) and size (from fewer than 200 to more than 2000 beds). At the healthcare-provider level, we recruited healthcare workers through convenience sampling depending on their roles in antibiotic prescribing in the selected 16 hospitals to participate in the knowledge, attitude, and practice (KAP) surveys and FGDs. In each hospital, we identified eligible staff available during the study period, who agreed to participate in the study and provided written informed consent. We conducted the national assessment between April 2022 and January 2023 through interviews and subsequent workshops with national stakeholders who held relevant positions and had expertise in AMR policy making. The G-ASET, KAP surveys and subsequent FGDs and interviews were conducted from December 2022 through June 2023.

We used the WHO national assessment tool from the WHO policy guidance on integrated AMS activities^1^ as the primary evaluation framework. This tool was selected for its comprehensive structure and alignment with WHO recommendations for evaluating AMS implementation within national AMR strategies. Other approaches (including the WHO Tracking Antimicrobial Resistance Country Self-Assessment Survey [TrACSS], national action plan for AMR [NAP-AMR] monitoring and evaluation, AWaRe [Access, Watch, Reserve] indicators) were considered complementary, as they focus on specific domains such as policy implementation, reporting, and antimicrobial consumption.

The tool consists of 44 questions categorised in five pillars and 12 domains, representing 12 packages of interventions to be considered and implemented to create a strong backbone for an integrated AMS approach to preserve antimicrobials at the national level. Each assessment item was scored as follows: 1 = No or not implemented; 2 = No, but a priority; 3 = Planned but not started; 4 = Partially implemented; 5 = Fully implemented.

For the facility-level assessment, the U.S. CDC's G-ASET was developed by a multidisciplinary group of physicians, pharmacists, microbiologists, and implementation scientists from different countries (including the U.S., Argentina, Viet Nam, Indonesia, Thailand, Nepal, Botswana, South Africa, Zimbabwe, and Switzerland) through a comprehensive process that included multiple rounds of review and revision, assembly of an external expert consensus panel using a Delphi-like approach, and international initial pilot testing (two sites in our network–general tertiary care hospital in Hanoi, Viet Nam in September 2022, and a community hospital in North Carolina, U.S. in October 2022). The newly developed G-ASET was used to assess AMS implementation at the hospital level in the participating sites in Asia. The modified G-ASET contained 68 closed-ended questions organised using the following five domains: 1) leadership commitment and accountability; 2) resources; 3) training and education; 4) AMS actions; and 5) antibiotic use tracking, monitoring and reporting.^15^ For most questions, the answers were formulated as not implemented (score 0), partially implemented (2.5), and fully implemented (5). For others, points were determined by how many answer choice options were selected. Semi-structured interviews were conducted among the participants who completed the national assessment tool and the modified G-ASET to obtain additional detail for each assessment item based on their responses.

At the individual level, a KAP survey form was developed concurrently with the G-ASET (following the same development and piloting process) to ascertain the knowledge, attitudes, and practices of healthcare workers (physicians, pharmacists, nurses, laboratory staff) regarding AMR, AMS, and antibiotic use (Supplementary File). The survey consisted of 41 closed-ended statements, and the assessment was based on a five-point Likert scale (from *strongly disagree* to *strongly agree*). Participants who were antibiotic prescribers were asked to complete all 41 questions, whereas non-prescribers responded to a subset of questions (questions 1–21).

We conducted FGDs with physicians and nurses who responded to the KAP survey to elicit in-depth insights into participants’ perceptions of AMR, antibiotic prescribing practices, and contextual factors influencing prescribing behaviours using a question guide (Supplementary File). Physicians and nurses were selected as they are central to antibiotic prescribing and use in clinical practice. The interviewers sent the national assessment form to the participants by email and asked them to complete it before the interview day. FGDs or interviews were held in a private location within each hospital, lasted approximately 1 h, and were conducted without the presence of non-participants.

The analysis of the national assessment survey comprised a review of initial responses given by interview participants, discussion and feedback given by workshop participants, and finalising the results with contextual information for each assessment item. A detailed assessment report for each country was prepared after the initial interviews and then reviewed by the interviewees and workshop participants to achieve a consensus on the current state of national-level AMS implementation. Here, we present the implementation status, measured by number of assessment items that have been partially or fully implemented under each domain by country as of 2023. We also summarised the main themes identified from these reports to describe the national context that is likely to be associated with facility-level implementation.

### Statistical analysis

We calculated the overall and domain-specific G-ASET scores by adding up the total points earned based on individual answers for each assessment question. The maximum possible scores by domain are: 65 for domain 1 (leadership commitment and accountability), 65 for domain 2 (resources), 25 for domain 3 (training and education), 105 for domain 4 (AMS actions), and 75 for domain 5 (tracking, monitoring, and reporting). We calculated a score by domain by summing the points earned for each question within a specific domain and then dividing by the maximum score per domain. We then summed the points earned across all domains and divided them by the total score of 335 to determine the overall level of AMS implementation achieved at each hospital.

We examined the correlations between the scores for specific domains and the overall score by calculating Spearman correlations and visualising as a correlation matrix. We also explored the associations between the overall G-ASET score and country, hospital type, and hospital size using a generalised linear model using R software (version 4.5.2). Hospital type was categorised as public or private, and hospital size was categorised as <500 beds, 500–1000 beds, and >1000 beds. Regression coefficients are reported as adjusted mean differences in total G-ASET score compared with the reference category. Given the small number of hospitals, the model was used as an exploratory analysis to identify possible associations.

We used descriptive analysis to describe KAP survey results across 16 hospitals (excluding those with missing data for specific KAP items), highlighting the areas to target for improvement.

We verbatim transcribed the audio recordings of the qualitative data from the interviews and FGDs and cross-checked the transcripts against field notes taken during the data collection. Following a thematic analysis method for identifying and analysing themes,^18^ transcripts were read by the research assistant at each site to generate initial categories following each question in the assessment forms (for interviews) and question guide (for FGDs). They were then reread in detail (by HYD and HTLV) to confirm whether categorisation was correct or if a new category was required (summarised in Microsoft Excel). Towards the end of the process, all transcripts were reviewed again to group responses into broader categories. Results were verified by the investigator team for validity and relevance.

While there was a pre-allocated number of interviews and FGDs at each site, data saturation was achieved at each hospital as little or no new information were obtained towards the last sessions in the assessment, allowing a comprehensive and in-depth understanding of the contexts for AMS implementation in the study hospitals. The quantitative results of each assessment item were sent back to the interviewers for confirmation of the content before data aggregation. The content of the qualitative findings was presented in the national- and hospital-level reports for feedback and corrections as needed. We integrated the qualitative interpretations into the quantitative findings in this manuscript to provide a cohesive representation of the current implementation state and its contextual factors to inform future improvement.

### Ethics statement

The research study was approved by the Oxford Tropical Research Ethics Committee (573-21), the Health Research Ethics Committee at Faculty of Medicine Universitas Indonesia (KET-334/UN2.F1/ETIK/PPM.00.02/2021), Nepal Health Research Council in Nepal (251/2022P), the Ethics Committee of the Faculty of Tropical Medicine, Mahidol University in Thailand (MUTM 2022-017-02), and the Institutional Review Board at the National Hospital for Tropical Diseases in Viet Nam (15-2022/HĐĐĐ-NĐTƯ). The Duke Institutional Review Board exempted the protocol for ethical review. This activity was reviewed by the U.S. CDC, deemed not research, and was conducted in accordance with applicable federal law and the U.S. CDC policy. Written informed consent was obtained from all participants prior to participation.

### Role of the funding source

The U.S. CDC solicited and funded the study and was involved in the conduct of the study; analysis and interpretation of the data; preparation, review, or approval of the manuscript; and decision to submit the paper for publication.

## Results

A total of 486 participants were included in this study, comprising stakeholders at the national level and healthcare workers at the facility level, including both frontline workers and leadership personnel. Of these, 118 were from Indonesia, 107 were from Nepal, 47 were from Thailand, and 214 from Viet Nam (Table 1). Of all participants, 13/486 (3%) participated in interviews for the national assessment, 84 (17%) in the workshops, 91 (19%) in the G-ASET and follow-up interviews, and 298 (61%) in the KAP survey (of which 134 also participated in 25 FGDs: 19 FGDs with physicians and six FGDs with nurses). All invited participants agreed to take part in the interviews, KAP survey, and FGDs. The distribution of participants varied across countries and assessment components, reflecting differences in participating hospitals, feasibility of data collection, and availability of respondents during the study period.

**Table 1:** Demographics of participants in the study in four countries, 2022–2023.

Assessment	Indonesia (n = 118)	Nepal (n = 107)	Thailand (n = 47)	Viet Nam (n = 214)	Total (n = 486)
National-level assessment (WHO assessment form)					
Interviews:					
Number of participants in interviews	1 (8)	6 (46)	4 (31)	2 (15)	13 (100)
Female participants in interviews	1 (12)	2 (25)	3 (38)	2 (25)	8 (100)
Workshops:					
Number of workshop participants	9 (11)	8 (9)	9 (11)	58 (69)^a^	84 (100)
Female participants in workshops	6 (12)	5 (10)	6 (12)	32 (66)	49 (100)
Participants with AMS leadership/coordination roles in workshops	6 (19)	3 (9)	2 (6)	21 (66)	32 (100)
Healthcare facility-level assessment (G-ASET)					
Number of participants in interviews^b^	28 (31)	18 (20)	9 (10)	36 (40)	91 (100)
Participants with AMS leadership roles	7 (33)	4 (19)	2 (10)	8 (38)	21 (100)
Participants who were physicians	10 (33)	5 (17)	3 (10)	12 (40)	30 (100)
Participants who were pharmacists	6 (35)	2 (12)	2 (12)	7 (41)	17 (100)
Participants who were microbiologists	4 (27)	4 (27)	0 (0)	7 (46)	15 (100)
Participants who were nurses or infection preventionists	1 (12)	3 (38)	2 (25)	2 (25)	8 (100)
Healthcare provider-level assessment					
Number of participants in KAP survey	80 (27)	75 (25)	25 (8)	118 (40)	298 (100)
Antibiotic prescribers	41 (25)	45 (27)	5 (3)	73 (45)	164 (100)
Physicians	43 (25)	43 (25)	5 (3)	80 (47)	171 (100)
Nurses	24 (27)	23 (26)	13 (14)	30 (33)	90 (100)
Pharmacists	9 (41)	1 (4)	7 (32)	5 (23)	22 (100)
Others	4 (27)	8 (53)	0 (0)	3 (20)	15 (100)
Number of participants in 25 FGDs	18 (13)	36 (27)	0 (0)	80 (60)	134 (100)
Physicians (participated in 19 FGDs)	11 (11)	36 (36)	0 (0)	53 (53)	100 (100)
Nurses (participated in 6 FGDs)	7 (21)	0 (0)	0 (0)	27 (79)	34 (100)

Data are presented as n (%). Percentages represent the distribution across countries within each row.

a Including one national and one regional workshop.

b Sex information was not collected from the participants in the healthcare-facility- and healthcare-provider-level assessments to maintain staff confidentiality, only staff roles were identified.

**Fig. 1:**
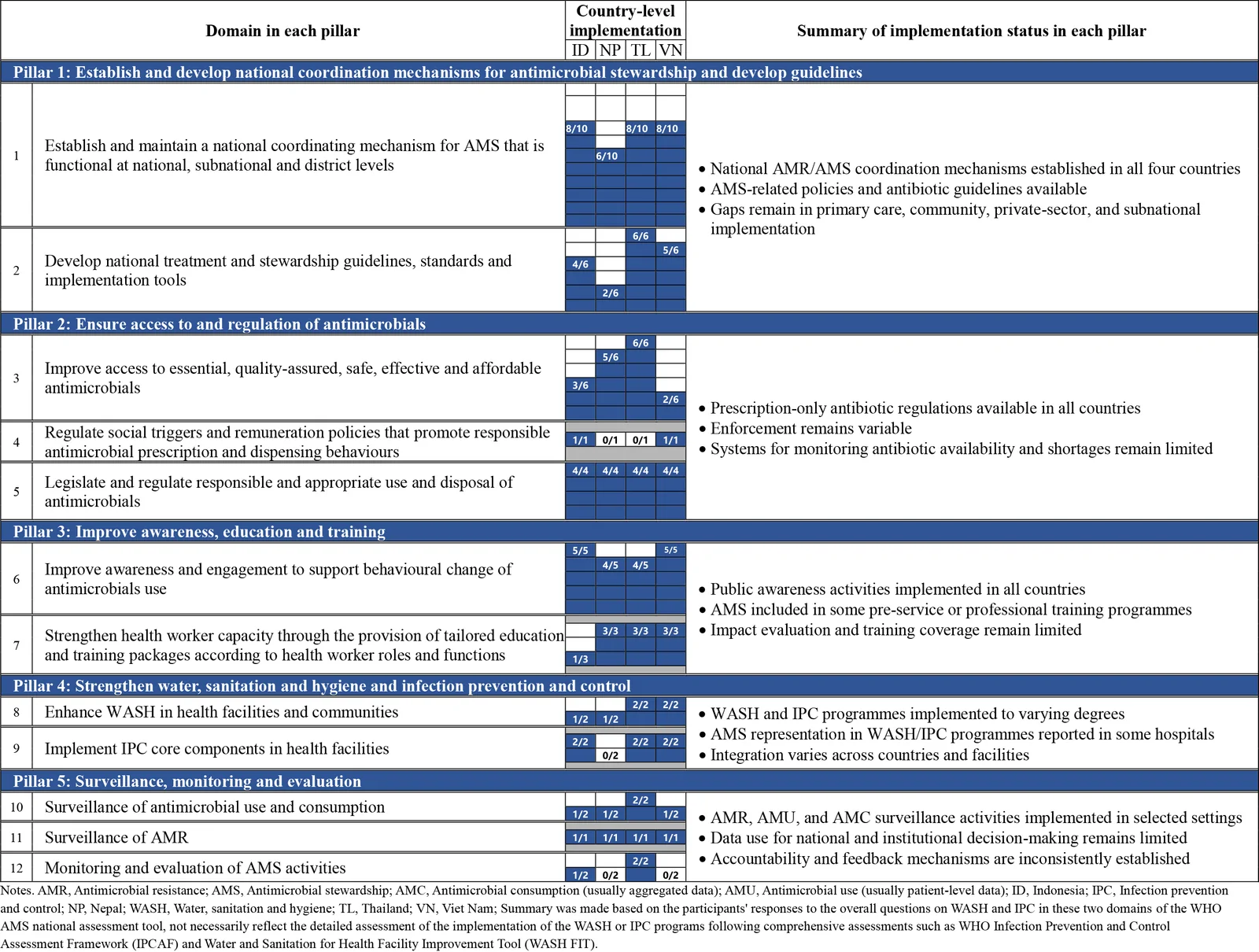
**Level of AMS implementation (*number of assessment items* that have been *partially or fully implemented* under each domain) by country, based on national assessment data collected during 2022–2023**.

At the national level, all four countries had taken meaningful steps towards establishing national coordinating mechanisms and guidance for AMR and AMS. The main observed gaps were a lack of comprehensive implementation across related sectors and programmes, varying levels of training for healthcare professionals, weak regulatory enforcement of prescription-only dispensing, and limited tracking mechanism for antibiotic shortages, and limited use of data for feedback and decision-making. While no overall funding mechanisms for AMR and AMS were identified in the national policies of Thailand and Viet Nam, there were specific budget allocations for these components included in the National Action Plan in Nepal and within the One Health framework in Indonesia. Fig. 1 summarises national-level AMS implementation at the time of assessment in 2023 for each pillar and its constituent domains corresponding to the WHO assessment tool. Details of the national-level context and implementation are provided in Tables S1 and S2 in the Supplementary File.

At the facility level, among 16 hospitals, the mean G-ASET score was 241/335 (72%), highest in tracking, monitoring, and reporting [56/75 (75%)] and lowest in training and education [17/25 (66%)] (Table 2). The overall G-ASET scores were highly correlated with the scores of the leadership commitment and accountability, AMS actions, and tracking, monitoring, and reporting domains, but not with the domains of resources and training and education (Figure S2). Resources and training and education were also poorly correlated with other domains in the G-ASET.

**Table 2:** Overall status of AMS implementation based on the G-ASET scores in each hospital at the time of assessment in four countries (2022–2023).

Hospital	Country	Type	Bed capacity	Overall score^a^		Leadership Commitment & Accountability	Resources	Training & Education	AMS actions	Tracking, Monitoring & Reporting
				(max: 335)		(max: 65)	(max: 65)	(max: 25)	(max:105)	(max: 75)
				score	%	%	%	%	%	%
H1	Indonesia	Private	<200	247.5	73.9	69.2	73.1	**80.0**	76.2	73.3
H2	Indonesia	Private	200–500	237.5	70.9	69.2	**84.6**	70.0	71.4	60.0
H3	Indonesia	Private	<200	170	50.7	53.8	73.1	10.0	47.6	46.7
H4	Indonesia	Private	200–500	274	**81.8**	**88.0**	65.0	**90.0**	**83.3**	**90.0**
H5	Indonesia	Public	500–1000	270	**80.6**	**84.6**	76.9	**100.0**	78.6	76.7
H6	Nepal	Public	<200	92.5	27.6	11.5	50.0	30.0	23.8	26.7
H7	Nepal	Private	200–500	262.5	78.4	**84.6**	73.1	60.0	**83.3**	76.7
H8	Nepal	Public	500–1000	155	46.3	46.2	65.4	20.0	42.9	43.3
H9	Thailand	Public	1000–1500	272.5	**81.3**	**88.5**	76.9	50.0	**83.3**	66.7
H10	Thailand	Public	500–1000	270	**80.6**	73.1	76.9	50.0	**83.3**	**96.7**
H11	Vietnam	Public	1000–1500	260	77.6	76.9	61.5	**90.0**	76.2	**90.0**
H12	Vietnam	Public	>2000	287.5	**85.8**	**96.2**	76.9	70.0	**81.0**	**96.7**
H13	Vietnam	Public	1000–1500	282.5	**84.3**	**96.2**	76.9	**90.0**	**81.0**	**83.3**
H14	Vietnam	Public	500–1000	275	**82.1**	76.9	73.1	70.0	**83.3**	**96.7**
H15	Vietnam	Public	200–500	232.5	69.4	73.1	61.5	**90.0**	59.5	**80.0**
H16	Vietnam	Public	1500–2000	270	**80.6**	76.9	69.2	**90.0**	**81.0**	**90.0**

a Scores are presented as absolute units achieved out of the maximum possible score of 335 and as percentages, calculated as the number of score units achieved divided by the total maximum score units for the overall score or the corresponding domain. Cells with scores ≥80% are highlighted in bold.

The crude mean G-ASET scores were similar across hospital types (238, 95% CI 188–289 in private hospitals versus 242, 95% CI 201–284 in public hospitals) but varied by hospital size. The adjusted estimates in the multivariable model suggested possible differences (Table 3). After adjustment for country, public hospitals had a lower mean G-ASET score than private hospitals, and larger hospitals had higher scores than smaller hospitals; however, these estimates were based on a small number of hospitals and should be interpreted as hypothesis-generating rather than definitive.

**Table 3:** Exploratory associations between hospital characteristics and G-ASET score among 16 hospitals in four countries (2022–2023).

Factor	N	Mean G-ASET score	95% CI	Difference^a^	95% CI^b^	p-value
Country						
Indonesia	5	240	188–292	reference		
Nepal	3	170	0–384	−33	−99 to 33	0.35
Thailand	2	271	255–287	57	−30 to 145	0.23
Viet Nam	6	268	247–289	68	−3 to 149	0.13
Type of hospital						
Private	5	238	188–289	reference		
Public	11	242	201–284	−100	−185 to −15	0.045
Hospital size						
<500 beds	7	217	157–276	reference		
500–1000 beds	4	242	150–335	74	3–146	0.071
>1000 beds	5	274	261–288	64	−10 to 137	0.12

a Difference in the G-ASET score of the corresponding subgroup compared to the reference subgroup in the multivariable generalised linear model.

b The 95% confidence interval (CI) corresponds to the lower and upper limit of the estimated difference. Crude mean G-ASET scores were slightly higher in public than private hospitals, whereas the adjusted model suggested a lower score for public hospitals; given the small sample size, this adjusted estimate should be interpreted cautiously.

Qualitative data showed that hospitals with higher scores in leadership commitment and accountability typically integrated AMS-specific activities into their annual plans, fostered multidisciplinary collaboration, and engaged in relevant external networking. Continuous training on AMS and IPC for hospital staff was partially delivered in 6/16 hospitals and fully delivered in 9/16 hospitals. Meanwhile, for AMS team, the training was partially delivered in 4/16 hospitals and fully delivered in 9/16 hospitals. A 24-h microbiology laboratory was available in 1/3 hospitals in Nepal and 13/13 hospitals in the other three countries. Access to Antimicrobial Use (AMU) and AMR electronic data was available in 15/16 hospitals; 5/16 hospitals had information and decision support systems to support AMS.

All hospitals, including those without a formal AMS programme, engaged in some AMS activities aimed at promoting prudent antibiotic prescribing, either hospital-wide or in selected wards because of resource constraints. Figure S3 also illustrates substantial variability in implementation across countries and between hospitals within the same country. Local antibiotic guidelines were developed in 1/3 hospitals in Nepal, and in all hospitals in other countries. The main gaps included limited use of antibiogram data to inform treatment guidelines (particularly in the hospitals in Nepal and Viet Nam), lack of standardised operating procedures for specific AMS activities (Indonesia and Nepal), absence of pre-authorisation policies (Indonesia and Nepal), and limited use of selective or cascading antibiotic susceptibility testing reports (Viet Nam).

For tracking and monitoring, 10/16 hospitals regularly conducted audits or point prevalence surveys to assess the appropriateness of antibiotic prescriptions (2/2 in Thailand, 6/6 in Viet Nam, 1/5 in Indonesia and 1/3 in Nepal). AMS teams in 10/16 hospitals reported AMS impact metrics to hospital leadership. In Nepal, 2/3 hospitals did not provide such reports due to the absence of an AMS team.

At the individual level, among 298 participants from 16 hospitals who undertook the KAP survey, 80 (27%) participants were from Indonesia, 75 (25%) from Nepal, 25 (8%) from Thailand and 118 (40%) from Viet Nam. 171 (57%) were physicians, 90 (30%) were nurses, and 22 (7%) were pharmacists. 164/298 (55%) were self-identified as antibiotic prescribers (Table S3). Overall, 117/292 participants (40%) agreed that antibiotics were overused; this was lower among non-prescribers (44/132, 33%) compared to prescribers (73/160, 46%). However, more non-prescribers (93/130, 70%) agreed there was multidisciplinary teamwork for antibiotic decision-making than prescribers (94/161, 58%). Similarly, more non-prescribers (93/132, 70%) also agreed that they were able to access the updated antibiogram than prescribers (97/161, 60%).

Among prescribers, training for healthcare professionals was deemed to be inadequate: 107/161 (66%) stated they received education on how to select the most appropriate antibiotic for treatment based on microbiology test results at their hospital. 100/161 (62%) agreed that they routinely obtained cultures before starting antibiotic therapy in patients with suspected infection, and 79/161 (49%) considered the opinion of non-physician staff in antibiotic decision-making (Table S3).

We sought to understand the contextual factors that could be associated with antibiotic prescribing among physicians and nurses who participated in 25 FGDs (19 among physicians and six among nurses) conducted in 11 hospitals in Indonesia, Nepal, and Viet Nam (FGDs were not feasible in Thailand). Five themes emerged from the FGDs across Indonesia, Nepal, and Viet Nam describing factors influencing day-to-day antibiotic prescribing practices (Table 4).

**Table 4:** Themes and representative quotes on the factors influencing antibiotic prescribing practices based on data collected during 2022–2023.

The colours indicate the qualitative salience of each theme, based on how frequently and strongly it was mentioned in the focus groups; they do not represent numerical scores. Red (+++) = major determinant, mentioned repeatedly across countries; dark yellow (++) = clearly important, but less consistently dominant; light yellow (+) = important, but mainly described in narrative form; blank = not mentioned in the FGDs in that country.

Abbreviations: ID, Indonesia, NP, Nepal; VN, Viet Nam.

Participants in Indonesia and Nepal emphasised the need to have clear antibiotic guidelines to support confident and appropriate decisions. However, they also noted that local antibiograms were often unavailable or not regularly updated, making it harder to choose appropriate empirical treatments.

Participants in Viet Nam described shortages of laboratory materials that delayed culture and sensitivity testing. In Nepal, doctors were concerned about unreliable specimen collection, unavailability of laboratory reagents, specifically the antimicrobial susceptibility testing discs for the antibiotics that are therapeutically used, and delays in reporting, particularly during holidays. Participants from Viet Nam also said that even when tests were completed, results were not always shared promptly with prescribers.

Doctors and nurses in Viet Nam reported frequent shortages of antibiotics and suitable antibiotic formulations, which often forced them to switch or compromise on treatment choices. These supply issues made it difficult to prescribe antibiotics appropriately. The limited availability of a comprehensive range of alternative antibiotic agents across multiple antibiotic classes was also reported by doctors in Nepal, hindering optimal treatment decision-making.

Participants described mixed experiences with antibiotic stewardship interventions. In Indonesia, antibiotic pre-authorisation was not yet implemented in the respective hospitals due to fears it might cause friction between AMS teams and prescribers. In contrast, nurses in Viet Nam reported that pharmacist-led pre-authorisation was already in place. In Nepal, only one of the three hospitals, which was a private facility with an AMS committee, reported implementing an antibiotic pre-authorisation process. Across all countries, participants mentioned the importance of teamwork and communication among AMS teams, clinicians, nurses, pharmacists, and microbiology staff. Nurses in Viet Nam said they were rarely involved in AMS activities but recognised their role in monitoring side effects, allergies, and raising patients’ awareness about antibiotics. They also expressed a need for more training to contribute effectively.

**Fig. 2:**
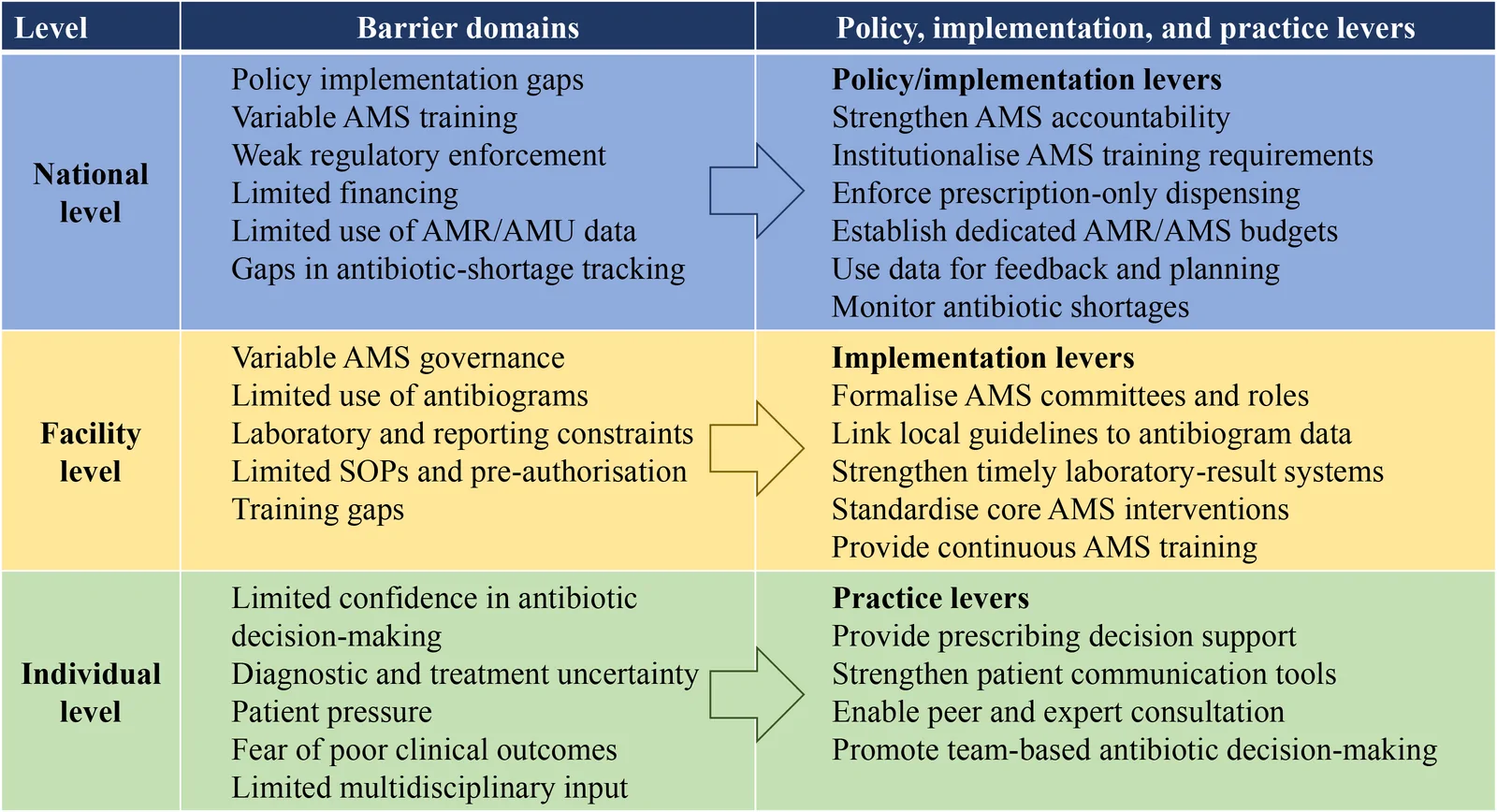
**Multilevel barriers and policy, implementation, and practice levers for strengthening antimicrobial stewardship implementation.** Barriers identified across national, facility, and individual levels are summarised alongside corresponding policy, implementation, and practice levers. AMS = antimicrobial stewardship. AMR = antimicrobial resistance. AMU = antimicrobial use. SOPs = standard operating procedures.

Doctors in Nepal and Viet Nam described pressure from patients to recover quickly and receive “strong” medicines. Doctors often prescribed antibiotics to meet these expectations or avoid complaints. Fear that patients could worsen or die if treatment failed also led some doctors to use broader-spectrum antibiotics or longer antibiotic courses than necessary. The main barriers to AMS implementation at three levels—national, facility, and individual—are summarised in Fig. 2, together with the possible levers for improvement.

## Discussion

Our findings indicate that although overall national coordination, guidance and policies for AMS were present in most countries, there was a lack of a comprehensive approach spanning different healthcare settings, with varying levels of implementation and support across countries. The national-level gaps included varying levels of training for healthcare professionals, weak regulatory enforcement of prescription-only dispensing, and limited tracking mechanism for antibiotic shortages, and limited use of data for feedback and decision-making. Consistently, findings at the facility and provider level also showed major gaps in training and use of local data to inform AMS actions.

National-level financing mechanisms for AMR control and AMS implementation are likely to have an impact on how specific activities and components are delivered at the local level.^19^ Similar to other low- and middle-income countries (LMICs),^11^ the lack of dedicated state funding was also highlighted in our study, particularly in Thailand and Viet Nam.

The G-ASET was useful for identifying major and nuanced areas for improvement. Leadership support was particularly important for coordinating AMS activities, enabling resource allocation, strengthening accountability, and embedding AMS into routine hospital structures. Nevertheless, implementation challenges remained common across the studied hospitals. The main gaps included a lack of updated treatment guidelines that incorporate local pathogen distribution and antimicrobial susceptibility patterns where available, and the absence of standardised operating procedures for specific AMS interventions. These gaps could be addressed through stronger national guidance and training. Guideline development and implementation tools are important for supporting resource-limited hospitals, as shown in a recent cluster randomised controlled trial in 16 district-level hospitals in Viet Nam.^20^ Recognising the need to support lower-level hospitals, the Viet Nam Ministry of Health recently issued a national handbook with recommendations tailored to the local context (Decision 2115/QD-BYT, 11 May 2023). The WHO Practical Toolkit for implementation of AMS programmes in healthcare facilities of LMICs and the U.S. CDC's Core Elements of Human Antibiotic Stewardship Programs in Resource-Limited Settings could also be used to guide context-relevant implementation.^21^^,^^22^ In addition, the WHO AWaRe (Access, Watch, Reserve) antibiotic book provides concise guidance on empiric antibiotic use for common clinical infections in primary care and community settings.^23^

AMS implementation also requires understanding of provider-level perceptions and awareness about AMR and AMS to tailor behaviour change approaches. In our convenience sample of KAP respondents from the four countries, 40% considered antibiotics to be overused in their facility. This is consistent with an earlier large survey administered among physicians administered in Indonesia in which 35% (357/1005) acknowledged that antibiotics were overused in their hospital while 86% agreed that antibiotics were overused in the country.^24^ Because the KAP survey was administered after the G-ASET interviews and focus groups, some responses may have been influenced by prior AMS discussions. This possible priming effect should be considered when interpreting the provider-level survey findings and comparisons with other surveys. Nonetheless, these findings suggest that AMS implementation in healthcare facilities in Asia could target improving recognition of antibiotic overuse as a problem among healthcare providers to support behaviour change in prescribing practices.

Nevertheless, barriers to appropriate antibiotic prescribing go beyond individuals' knowledge and attitudes, as shown in our KAP survey and qualitative findings. At the provider level, prescribing decisions were also shaped by patient-related factors, including expectations for rapid recovery, requests for antibiotics, and prescribers’ fear of treatment failure, which often led to broader-spectrum or longer antibiotic courses than clinically necessary. Previous studies have similarly shown the need for AMS implementation approaches that take the sociocultural context of prescribing into account.^25^^,^^26^ In our study, the respondents also reported the need to improve the effectiveness of multidisciplinary teams and to better acknowledge the views of non-physician staff, such as pharmacists in antibiotic decision-making. One important barrier was the limited implementation of pre-authorisation policies for selected antibiotics, which participants linked to friction between AMS teams and prescribers. This highlights that effective AMS requires not only clear rules and oversight, but also sustained interprofessional communication, shared ownership, and locally acceptable stewardship processes. Context-adapted stewardship, with shared responsibility across the healthcare team rather than reliance on individual prescribers alone, has been suggested,^27^^,^^28^ and may help optimise antibiotic use while making better use of scarce resources. Further challenges could arise from system-related factors, including limited access to antibiotics, which may lead to inappropriate selection of antibiotics. Sustainable access to antibiotics is therefore critical for AMS implementation and requires national-level financial support.

While the strengths of this study include the participation of different types of hospitals in two upper-middle-income and two lower-middle income countries with different levels of economic and development status and an assessment approach integrated findings across different levels of AMS implementation, we recognise important limitations. First, the small sample size of hospitals and respondents, combined with convenience sampling through local research networks, limits the generalisability of the findings, which may not reflect nationwide AMS implementation. The provider-level qualitative findings reflect perceptions from the three countries where FGDs were conducted and should be interpreted within that sampling context. Second, some components of the assessments were based on self-reporting, which has the potential to overestimate achievements in AMS implementation. Because participation was voluntary and data were self-reported, some degree of selection and social desirability bias may have influenced responses. We did not systematically verify all reported practices through independent observation or routine audit data across all sites, so the findings should be interpreted as participant-reported perceptions and experiences of AMS implementation. Third, some sensitive factors influencing AMS implementation, such as pharmaceutical industry incentives, may not have been fully captured despite the mixed-methods design and non-threatening data collection approach. Finally, the use of a single framework for the national-level assessment may not capture all dimensions of AMS implementation, particularly antimicrobial consumption and participant-reported national progress.

This mixed-methods study identified substantial variability in AMS implementation across countries and hospitals, and highlighted important gaps in national guidance, hospital-level systems, use of microbiology data, training, and multidisciplinary collaboration. At the provider level, prescribing was shaped not only by knowledge and attitudes, but also by patient demand, fear of treatment failure, and constraints in antibiotic availability and microbiology services. These findings support context-specific AMS approaches that combine national guidance and financing with stronger hospital-level implementation, locally informed protocols, and support for smaller hospitals and primary care settings. They also suggest that AMS strategies should address patient expectations and communication around antibiotic use, in addition to prescriber behaviour and hospital systems. As an exploratory cross-sectional study, these findings highlight priority areas for improvement.

## Contributors

HTLV, RLH, DL, AK, EDA, DJA, and HRvD contributed to conceptualisation, methodology, and funding acquisition. RL, SM, DL, HYD, and HTLV contributed to data curation. RL, SM, DL, HYD, and HTLV contributed to formal analysis. Investigation was carried out by all authors in the G-ASET Development and Implementation Group in Asia, which supported the study design, coordination, and implementation across participating countries. RL and HTLV contributed to writing—original draft. All authors contributed to writing—review & editing and finalised the manuscript. DL, AA, RLH, EJN, AK, VGT, HRvD, and HTLV contributed to supervision. All authors had access to all the data reported in the study. RL and HTLV accessed and verified the underlying data. All authors read and approved the final version.

## Data sharing statement

All the data in the manuscript have been provided in the manuscript and the Supplementary Material.

## Declaration of interests

We declare no competing interests.

## Appendix A Supplementary data

Supplementary Material

Translated Summary
