# Supplementary material for: Factors influencing antimicrobial stewardship implementation in healthcare facilities in four countries of Southeast Asia: a mixed-methods study (2022–23)
Source: Lancet Reg Health Southeast Asia. 2026 Aug 6;53:100837. doi: 10.1016/j.lansea.2026.100837 (PMC13546922; doi:10.1016/j.lansea.2026.100837)
Supplement: Supplementary file 1 [file mmc1.docx]

**Supplemental File**

**Contextual factors associated with antimicrobial stewardship implementation in healthcare facilities: a four-country mixed-methods study in South and Southeast Asia**

**Authors:** Ralalicia Limato, MD‎^1,2^, Sulochana Manandhar, PhD^3^, Professor Direk Limmathurotsakul, PhD^2,4^‎, Professor Anucha Apisarnthanarak, MD^5^, Raph L. Hamers, MD^1,2^‎, Professor Erni J. Nelwan, MD^6,7^, Abhilasha Karkey, PhD^2,3^, Twisha S. Patel, PharmD^8^, Fernanda C. Lessa, MD^8^, Professor Elizabeth Dodds Ashley, PharmD^9^, Professor Deverick J. Anderson, MD^9^, Duong Hai Yen, MSc^10^, Tran Van Giang, MD^11,12^, Professor H Rogier van Doorn, PhD^2,3^, Vu Thi Lan Huong, PhD^10*^, on behalf of the **G-ASET Development and Implementation Group in Asia**.

^1^Oxford University Clinical Research Unit Indonesia, Faculty of Medicine Universitas Indonesia, Jakarta, Indonesia

^2^Centre for Tropical Medicine and Global Health, Nuffield Department of Medicine, University of Oxford, Oxford, UK

^3^Oxford University Clinical Research Unit Nepal, Kathmandu, Nepal

^4^Mahidol Oxford Tropical Medicine Research Unit, Faculty of Tropical Medicine, Mahidol University, Bangkok, Thailand

^5^Faculty of Medicine, Thammasat University Hospital, Pratumthani, Thailand

^6^Faculty of Medicine, Universitas Indonesia, Jakarta, Indonesia

^7^Division of Infectious Diseases, Department of Internal Medicine, Cipto Mangunku- sumo National General Hospital, Jakarta, Indonesia

^8^Division of Healthcare Quality Promotion, U.S. Centers for Disease Control and Prevention, United States

^9^Duke Antimicrobial Stewardship Outreach Network, Duke Center for Antimicrobial Stewardship and Infection Prevention, Duke University, Durham, NC 27710, United States

^10^Oxford University Clinical Research Unit Viet Nam, Thon Bau, Thien Loc, Hanoi, Viet Nam

^11^Hanoi Medical University, No. 1 Ton That Tung, Kim Lien, Hanoi, Viet Nam

^12^National Hospital for Tropical Diseases, Thon Bau, Thien Loc, Hanoi, Viet Nam

***Corresponding author:** Vu Thi Lan Huong, email: [huongvtl@oucru.org](mailto:huongvtl@oucru.org), telephone: + 84 24 35764320; fax : +84 24 35764319

**Supplemental material. Details of the G-ASET Development and Implementation Group in Asia**

| **Name/ Institution** | **Surname** | **Firstname** | **Degree** | **Role** |
| --- | --- | --- | --- | --- |
| **Indonesia** | | | | |
| **OUCRU Indonesia** | | | | |
| Raph L. Hamers | Hamers | Raph L | MD | Country PI |
| Ralalicia Limato | Limato | Ralalicia | MD | Post-doctoral researcher, conducting interviews (female) |
| Elza Samantha Elmira | Elmira | Elza Samantha | MPH | Research Assistant, conducting interviews (female) |
| Fariz R. Iswoyo | Iswoyo | Fariz R | MD | Research Assistant |
| Ivan Damara | Damara | Ivan | MD | Research Assistant, conducting interviews and FGDs (male) |
| Korrie Salsabila | Salsabila | Korrie | M.Biomed | Research Assistant |
| Marita Joesoef | Joesoef | Marita | BSc | Research Assistant |
| Muhammad R. H. Lubis | Lubis | Muhammad RH | MD | Research Assistant |
| Talitha V. Salsabella | Salsabella | Talitha V | MD | Research Assistant |
| Tamara Tango | Tango | Tamara | MD | Research Assistant |
| Taris Radifan | Radifan | Taris | MD | Research Assistant |
| **Royal Taruma Hospital, Jakarta, Indonesia** | | | | |
| Enty Tjoa | Tjoa | Enty | MD | Site investigator |
| **MMC Hospital, Jakarta, Indonesia** | | | | |
| Erni Juwita Nelwan | Nelwan | Erni Juwita | MD | Site investigator |
| **RS Carolus, Jakarta, Indonesia** | | | | |
| Robert Sinto | Sinto | Robert | MD | Site investigator |
| **RS Islam Cempaka Putih, Jakarta, Indonesia** | | | | |
| Sharifah Shakinah | Shakinah | Sharifah | MD | Site investigator |
| **Dr. Cipto Mangunkusumo National General Hospital, Jakarta, Indonesia** | | | | |
| Erni Juwita Nelwan | Nelwan | Erni Juwita | MD | Site investigator |
| **Nepal** | | | | |
| **OUCRU Nepal** | | | | |
| Abhilasha Karkey | Karkey | Abhilasha | PhD | Country PI |
| Sulochana Manandhar | Manandhar | Sulochana | PhD | Post doctoral researcher, conducting interviews and FGDs (female) |
| Sushmita Shrestha | Shrestha | Sushmita | BSc | Research Assistant, conducting interviews and FGDs (female) |
| Alisha Pradhan | Pradhan | Alisha |  | Study coordination |
| **Grande International Hospital, Dhapasi, Kathmandu, Nepal** | | | | |
| Binod Bijukchhe | Bijukchhe | Binod | MD | Site investigator |
| Prashant Tripathi | Tripathi | Prashant | MD | Site investigator, study coordination |
| **B.P. Koirala Institute of Health Sciences, Dharan, Kathmandu, Nepal** | | | | |
| Basudha Khanal | Khanal | Basudha | MD | Site investigator, study coordination |
| Sanjib Kumar Sharma | Sharma | Sanjib Kumar | MD | Site investigator |
| Narayan Raj Bhattarai | Bhattarai | Narayan Raj | MD | Site investigator |
| Ratna Baral | Baral | Ratna | MD | Site investigator |
| Shyam Prasad Kafle | Kafle | Shyam Prasad | MD | Site investigator |
| Piyush Dahal | Dahal | Piyush | MSc | Research Assistant, conducting interviews and FGDs |
| **Nepal Korea Friendship Municipality Hospital, Thimi, Bhaktapur, Nepal** | | | | |
| Dipendra Kumar Raushan | Raushan | Dipendra Kumar | MD | Site investigator |
| Gyan Kayastha | Kayastha | Gyan | MD | Site investigator |
| Maya Bade | Bade | Maya | MSc | Study coordination |
| **Thailand** | | | | |
| **Mahidol Oxford Tropical Medicine Research Unit - Bangkok, Thailand** | | | | |
| Direk Limmathurotsakul | Limmathurotsakul | Direk | PhD | Country PI; conducting interviews (national) (male) |
| Pornpan Sunthornsut | Sunthornsut | Pornpan | BSc | Research assistant, conducting interviews and FGDs (hospital) (female) |
| **Faculty of Medicine, Thammasat University Hospital, Pratumthani, Thailand** | | | | |
| Anucha Apisarnthanarak | Apisarnthanarak | Anucha | MD | Co-investigator |
| **Department of Medical Services, Ministry of Public Health, Thailand** | | | | |
| Amornrat Vijitleela | Vijitleela | Amornrat | PH | National stakeholder |
| **Food and Drug Administration, Ministry of Public Health, Thailand** | | | | |
| Nuntiya Somjetanakul | Somjetanakul | Nuntiya | PH | National stakeholder |
| **International Health Policy Program (IHPP), Thailand** | | | | |
| Anond Kulthanmanusorn | Kulthanmanusorn | Anond | MD | National stakeholder |
| **Bamrasnaradura Infectious Disease Institute, Thailand** | | | | |
| Lantharita Charoenpong | Charoenpong | Lantharita | MD | National stakeholder |
| **Chiangrai Prachanukroh Hospital, Ubon Ratchathani, Thailand** | | | | |
| Suwimon Khusuwan | Khusuwan | Suwimon | MD | Site-investigator |
| Suchada Ruenglertphong | Ruenglertphong | Suchada | Pediatric ID | Pediatric ID |
| Phiradee Chanthirawong | Chanthirawong | Phiradee |  | Pharmacist |
| Patcharin Sumanapunyawong | Sumanapunyawong | Patcharin |  | Infection Control Nurse |
| Sureeruk Achalapong | Achalapong | Sureeruk |  | Infection Control Nurse |
| Suwatthiya Kitsaran | Kitsaran | Suwatthiya | MD | Site-investigator |
| Patcharee Karnjanawat | Karnjanawat | Patcharee |  | Pharmacist |
| Korawan Pudpong | Pudpong | Korawan |  | Pharmacist |
| Ramphai Sukkasem | Sukkasem | Ramphai |  | Infection Control Nurse |
| Somsamai Boonsong | Boonsong | Somsamai |  | Infection Control Nurse |
| **Viet Nam** | | | | |
| **OUCRU Viet Nam** | | | | |
| Vu Thi Lan Huong | Vu | Huong TL | PhD | Principal investigator |
| H Rogier van Doorn | van Doorn | H Rogier | PhD | Co-investigator |
| Duong Hai Yen | Duong | Hai Yen | MSc | Research Assistant, conducting interviews and FGDs (female) |
| Phan Thi Thu Hien | Phan | Thi Thu Hien | MSc | Study Coordinator |
| Nguyen Thi Cam Tu | Nguyen | Thi Cam Tu | MSc | PhD student, conducting interviews (female) |
| Truong Anh Quan | Truong | Anh Quan | MSc | Research Assistant |
| Nguyen Hai Yen | Nguyen | Hai Yen | MSc | Research Assistant |
| **National Hospital for Tropical Diseases, Hanoi, Viet Nam** | | | | |
| Nguyen Thanh Ha | Nguyen | Thanh Ha | MD | Site investigator |
| Pham Ngoc Thach | Pham | Ngoc Thach | MD | Hospital leader |
| Nguyen Van Cap | Nguyen | Van Cap | MD | Hospital leader |
| Tran Van Giang | Tran | Van Giang | MD | Hospital study coordinator |
| Le Nguyen Minh Hoa | Le | Nguyen Minh Hoa | PhD | Hospital study coordinator |
| **Huu Nghi Hospital, Hanoi, Viet Nam** | | | | |
| Pham Thi Thuy Van | Pham | Thi Thuy Van | PhD | AMS Lead, Clinical Pharmacy |
| Nguyen Thi Thu Huong | Nguyen | Thi Thu Huong |  | Site coordinator |
| Dinh Thi Chi | Dinh | Thi Chi |  | Research Team |
| **Dong Da District Hospital, Hanoi, Viet Nam** | | | | |
| Pham Ba Hien | Pham | Ba Hien | MD | Hospital leader |
| Nguyen Thi Phuong Thuy | Nguyen | Thi Phuong Thuy | MD | Site investigator |
| Nguyen Thu Hang | Nguyen | Thu Hang |  | Site coordinator |
| **Vietnam – Sweden Uong Bi Hospital, Quang Ninh** | | | | |
| Tran Anh Cuong | Tran | Anh Cuong | MD | Hospital leader |
| Nguyen Thi Ngoc Diep | Nguyen | Thi Ngoc Diep | MD | Site investigator |
| Nguyen Thi Thuy | Nguyen | Thi Thuy |  | Site coordinator |
| **Nghe An Provincial Hospital, Nghe An, Viet Nam** | | | | |
| Trinh Xuan Nam | Trinh | Xuan Nam | MD | Hospital leader |
| Nguyen Ngoc Hoa | Nguyen | Ngoc Hoa | MD | Site investigator |
| Tran Anh Dao | Tran | Anh Dao |  | Site coordinator |
| **Hue Central Hospital, Hue, Viet Nam** | | | | |
| Hoang Thi Lan Huong | Hoang | Thi Lan Huong | MD | Site investigator |
| Mai Van Tuan | Mai | Van Tuan | MD | Site coordinator |
| **Dong Thap General Hospital, Cao Lanh, Dong Thap, Viet Nam** | | | | |
| Chau Minh Duc | Chau | Minh Duc | MD | Hospital leader, Site investigator |
| Vo Thi Hoang Dung Em | Vo | Thi Hoang Dung Em | MD | Site coordinator |
| **Medical Services Administration, Ministry of Health, Viet Nam** | | | | |
| Cao Hung Thai | Cao | Hung Thai | PhD | National stakeholder |
| **Wilson Medical Center, Wilson, North Carolina, United States** | | | | |
| Christian Shaw | Shaw | Christian | PharmD | Stewardship Pharmacist |
| **Duke Antimicrobial Stewardship Outreach Network, Duke University, Durham, United States** | | | | |
| Deverick J Anderson | Anderson | Deverick J | MD, MPH | Co-investigator |
| Elizabeth Dodds Ashley | Dodds Ashley | Elizabeth | PharmD, MHS | Co-investigator |
| **Division of Healthcare Quality Promotion, US Centers for Disease Control and Prevention, United States** | | | | |
| Fernanda C. Lessa | Lessa | Fernanda C | MD | Co-investigator |
| Twisha S. Patel | Patel | Twisha S | PharmD | Co-investigator |

**Supplemental Figure S1. Timeline and sequence of data collection methods in the study during 2022-2023**

**Supplemental material. KAP survey form**

Developed as part of the development of AMS assessment tools conducted under a U.S. Centers for Disease Control and Prevention (U.S. CDC)-led global initiative, aiming to assess AMS implementation in healthcare facilities. The KAP form was concurrently developed and piloted together with The Global Antibiotic Stewardship Evaluation Tool (G-ASET) for Inpatient Healthcare Facilities across Asia, Latin America, and Southern Africa.^15^

**Antibiotic Stewardship Survey for Healthcare Workers**

**This survey** assesses the **knowledge, attitudes, and practices of various healthcare workers (e.g., physicians, pharmacists, nurses)** regarding antibiotic use, antibiotic stewardship, and antibiotic resistance.

**Please select your healthcare profession:** ☐ physician ☐ pharmacist ☐ nursing staff (e.g., nurse, nurse technician) ☐ other, please specify:

**Are you currently in training (e.g., residency, fellowship)?** ☐ yes ☐ no

**Please enter the number of years of experience that you have in the selected healthcare profession.** _____

**Please enter the number of years that you have worked at this healthcare facility.** ______

**Do you prescribe antibiotics?** ☐ yes ☐ no (If ***yes***, please rate your agreement with **ALL** items. If ***no***, please rate your agreement with items **1-21**).

| **FOR *ALL* HEALTHCARE WORKERS: Please rate your agreement with each of the following statements below.** | | | **Notes/Comments** |
| --- | --- | --- | --- |
| 1 | Optimizing antibiotic use is a priority at my healthcare facility. | ☐ strongly agree  ☐ agree  ☐ neutral  ☐ disagree  ☐ strongly disagree  ☐ not applicable |  |
| 2 | Antibiotic use is discussed at facility-wide multidisciplinary team meetings. | ☐ strongly agree  ☐ agree  ☐ neutral  ☐ disagree  ☐ strongly disagree  ☐ not applicable |  |
| 3 | I am familiar with the term antibiotic stewardship. | ☐ strongly agree  ☐ agree  ☐ neutral  ☐ disagree  ☐ strongly disagree  ☐ not applicable |  |
| 4 | The importance of antibiotic stewardship is communicated (e.g., via posters, emails) at my healthcare facility. | ☐ strongly agree  ☐ agree  ☐ neutral  ☐ disagree  ☐ strongly disagree  ☐ not applicable |  |
| 5 | I trust the microbiology test results that I receive at my healthcare facility. | ☐ strongly agree  ☐ agree  ☐ neutral  ☐ disagree  ☐ strongly disagree  ☐ not applicable |  |
| 6 | My healthcare facility promptly alerts prescribers about relevant positive culture results (e.g., organism identified in blood culture) to modify antibiotic therapy. | ☐ strongly agree  ☐ agree  ☐ neutral  ☐ disagree  ☐ strongly disagree  ☐ not applicable |  |
| 7 | I am able to access my healthcare facility's updated antibiogram. | ☐ strongly agree  ☐ agree  ☐ neutral  ☐ disagree  ☐ strongly disagree  ☐ not applicable |  |
| 8 | More guidance from the antibiotic stewardship team could improve antibiotic use at my healthcare facility. | ☐ strongly agree  ☐ agree  ☐ neutral  ☐ disagree  ☐ strongly disagree  ☐ not applicable |  |
| 9 | Use of broad-spectrum antibiotics when equally effective narrower spectrum antibiotics are available increases antibiotic resistance. | ☐ strongly agree  ☐ agree  ☐ neutral  ☐ disagree  ☐ strongly disagree  ☐ not applicable |  |
| 10 | Inappropriate antibiotic use can harm patients. | ☐ strongly agree  ☐ agree  ☐ neutral  ☐ disagree  ☐ strongly disagree  ☐ not applicable |  |
| 11 | The incidence of antibiotic-resistant organisms can be reduced by optimizing antibiotic prescribing patterns and infection prevention and control practices. | ☐ strongly agree  ☐ agree  ☐ neutral  ☐ disagree  ☐ strongly disagree  ☐ not applicable |  |
| 12 | Appropriate use of antibiotics may reduce antibiotic resistance. | ☐ strongly agree  ☐ agree  ☐ neutral  ☐ disagree  ☐ strongly disagree  ☐ not applicable |  |
| 13 | Requiring clinicians to obtain approval prior to prescribing certain antibiotics is a way to improve antibiotic use. | ☐ strongly agree  ☐ agree  ☐ neutral  ☐ disagree  ☐ strongly disagree  ☐ not applicable |  |
| 14 | Antibiotics are overused (e.g., antibiotics are used when not clinically indicated) at my healthcare facility. | ☐ strongly agree  ☐ agree  ☐ neutral  ☐ disagree  ☐ strongly disagree  ☐ not applicable |  |
| 15 | Antibiotic resistance is a problem at my healthcare facility. | ☐ strongly agree  ☐ agree  ☐ neutral  ☐ disagree  ☐ strongly disagree  ☐ not applicable |  |
| 16 | There is multidisciplinary teamwork for antibiotic decision-making activities at my healthcare facility. | ☐ strongly agree  ☐ agree  ☐ neutral  ☐ disagree  ☐ strongly disagree  ☐ not applicable |  |
| 17 | I value recommendations from the antibiotic stewardship team at my healthcare facility. | ☐ strongly agree  ☐ agree  ☐ neutral  ☐ disagree  ☐ strongly disagree  ☐ not applicable |  |
| 18 | I have access to locally endorsed infectious diseases treatment guidelines used at my healthcare facility. | ☐ strongly agree  ☐ agree  ☐ neutral  ☐ disagree  ☐ strongly disagree  ☐ not applicable |  |
| 19 | I have adequate access to infectious diseases expertise (e.g., infectious diseases trained physician or clinician with experience practicing infectious diseases) at my healthcare facility. | ☐ strongly agree  ☐ agree  ☐ neutral  ☐ disagree  ☐ strongly disagree  ☐ not applicable |  |
| 20 | I feel comfortable recommending an intervention to my colleagues on antibiotic use. | ☐ strongly agree  ☐ agree  ☐ neutral  ☐ disagree  ☐ strongly disagree  ☐ not applicable |  |
| 21 | Healthcare workers (e.g., physicians, pharmacists, or nurses) educate patients and/or their families on the use of antibiotics at discharge at my healthcare facility. | ☐ strongly agree  ☐ agree  ☐ neutral  ☐ disagree  ☐ strongly disagree  ☐ not applicable |  |

| **FOR *PRESCRIBERS* ONLY: Please rate your agreement with each of the following statements below.** | | | **Notes/Comments** |
| --- | --- | --- | --- |
|  | | |  |
| 22 | I have timely access to microbiology test results and diagnostic information to guide my use of antibiotics. | ☐ strongly agree  ☐ agree  ☐ neutral  ☐ disagree  ☐ strongly disagree  ☐ not applicable |  |
| 23 | Receiving more education on appropriate selection of antibiotic agent, duration of therapy, and dose could improve my antibiotic prescribing practices at my healthcare facility. | ☐ strongly agree  ☐ agree  ☐ neutral  ☐ disagree  ☐ strongly disagree  ☐ not applicable |  |
| 24 | I receive education on how to select the most appropriate antibiotic for treatment based on microbiology test results at my healthcare facility. | ☐ strongly agree  ☐ agree  ☐ neutral  ☐ disagree  ☐ strongly disagree  ☐ not applicable |  |
| 25 | The antibiotic stewardship team can impact my decisions on antibiotic initiation and continuation at my healthcare facility. | ☐ strongly agree  ☐ agree  ☐ neutral  ☐ disagree  ☐ strongly disagree  ☐ not applicable |  |
| 26 | I am pressured to prescribe antibiotics by patients or their families. | ☐ strongly agree  ☐ agree  ☐ neutral  ☐ disagree  ☐ strongly disagree  ☐ not applicable |  |
| 27 | I am pressured to prescribe antibiotics by my colleagues. | ☐ strongly agree  ☐ agree  ☐ neutral  ☐ disagree  ☐ strongly disagree  ☐ not applicable |  |
| 28 | Scientific literature (e.g., published research) influences my decisions on antibiotic prescribing at my healthcare facility. | ☐ strongly agree  ☐ agree  ☐ neutral  ☐ disagree  ☐ strongly disagree  ☐ not applicable |  |
| 29 | Pharmaceutical companies influence some of my decisions on antibiotic prescribing at my healthcare facility. | ☐ strongly agree  ☐ agree  ☐ neutral  ☐ disagree  ☐ strongly disagree  ☐ not applicable |  |
| 30 | I use locally endorsed infectious diseases treatment guidelines when I am making decisions about antibiotic prescribing at my healthcare facility. | ☐ strongly agree  ☐ agree  ☐ neutral  ☐ disagree  ☐ strongly disagree  ☐ not applicable |  |
| 31 | I prescribe certain empiric antibiotics based on consultation with a clinician with experience practicing infectious diseases, infectious diseases trained physician, or the antibiotic stewardship team at my healthcare facility. | ☐ strongly agree  ☐ agree  ☐ neutral  ☐ disagree  ☐ strongly disagree  ☐ not applicable |  |
| 32 | I routinely obtain cultures before starting antibiotic therapy in patients with suspected infection at my healthcare facility. | ☐ strongly agree  ☐ agree  ☐ neutral  ☐ disagree  ☐ strongly disagree  ☐ not applicable |  |
| 33 | I modify my patient's antibiotic treatment after receiving culture and antibiotic susceptibility results when appropriate. | ☐ strongly agree  ☐ agree  ☐ neutral  ☐ disagree  ☐ strongly disagree  ☐ not applicable |  |
| 34 | I consider adverse events when selecting an antibiotic regimen for patients at my healthcare facility. | ☐ strongly agree  ☐ agree  ☐ neutral  ☐ disagree  ☐ strongly disagree  ☐ not applicable |  |
| 35 | I consider drug interactions when selecting an antibiotic regimen for a defined patient population at my healthcare facility. | ☐ strongly agree  ☐ agree  ☐ neutral  ☐ disagree  ☐ strongly disagree  ☐ not applicable |  |
| 36 | I consider my patient's kidney function when dosing antibiotics at my healthcare facility. | ☐ strongly agree  ☐ agree  ☐ neutral  ☐ disagree  ☐ strongly disagree  ☐ not applicable |  |
| 37 | I consider the risk of development of antibiotic resistance in my patients when I prescribe antibiotics. | ☐ strongly agree  ☐ agree  ☐ neutral  ☐ disagree  ☐ strongly disagree  ☐ not applicable |  |
| 38 | I consider the opinion of non-physician staff (e.g., nursing, pharmacy) in antibiotic decision-making at my healthcare facility. | ☐ strongly agree  ☐ agree  ☐ neutral  ☐ disagree  ☐ strongly disagree  ☐ not applicable |  |
| 39 | Receiving feedback about appropriateness of antibiotics that I prescribe could improve my antibiotic prescribing practices. | ☐ strongly agree  ☐ agree  ☐ neutral  ☐ disagree  ☐ strongly disagree  ☐ not applicable |  |
| 40 | Receiving feedback on how my antibiotic prescribing practices compares to my peers could improve my antibiotic prescribing practices. | ☐ strongly agree  ☐ agree  ☐ neutral  ☐ disagree  ☐ strongly disagree  ☐ not applicable |  |
| 41 | I am aware of changes that are needed to my current antibiotic prescribing practices based on feedback received at my healthcare facility. | ☐ strongly agree  ☐ agree  ☐ neutral  ☐ disagree  ☐ strongly disagree  ☐ not applicable |  |

**Supplemental material. Question guide for FGD at the healthcare facilities**

|  | **Question** |
| --- | --- |
| **OPENING**  (5 minutes) | 1. Tell us who you are, what your background by training is, how long you have been working for this hospital, what your current work position is, and how long you have been in this position for at this hospital. |
| **INTRODUCTION**  (10 minutes) | 2. What is the first thing that comes to mind when you hear the word “antibiotic resistance”? Why is this important? What are the reasons for antibiotic resistance at your hospital? |
| **KEY**  (60 minutes) | 3. How often are patients in your department prescribed antibiotics? What are the main activities do you often do in patients with antibiotic prescription? What are the challenges and difficulties you often encounter in these activities (probing: in terms of decision making on when and how to initiate, change or stop antibiotics, caring for patients with antibiotic prescriptions, support from hospital for this work…) 4. Have you heard of the antimicrobial stewardship (AMS) program? Please tell us what you understand about this program? Do you know about any guidelines or policy used at your hospital that are related to AMS? 5. What are the actions that you are aware of having been implemented as part of the AMS program in this hospital? (if needed, promptin by referring to the specific actions as listed in the Supplemental Table B of G-ASET form) 6. What are the resources available to you to access in relation to care and treatment of patients in patients who need antibiotic prescription? (if needed prompting by mentioned about AMS team support, guidelines, drugs and drug expertise, infection management expertise, microbiology services, antibiogram) 7. What are the strengths of and opportunities for AMS implementation at your hospital? 8. What are the weaknesses of and barriers for AMS implementation at your hospital? 9. What do you think you can do to help improve antibiotic use at your hospital? |
| **ENDING**  (5 minutes) | *Moderator summarizes the main content of the focus group.*   10. Is there anything else you would like to add or mention before we wrap- up? |

**Supplemental Table S1. Summary of national contexts for AMS implementation in Indonesia, Nepal, Thailand and Viet Nam based on collected national assessment data, 2022-2023**

| Country^1^ | National contexts for AMS implementation^2^ |
| --- | --- |
| Indonesia  Population: approximately 284 million;  Population density: 143/km^2^  Gross Domestic Product (nominal) per capita: $5,027  Human Development Index: 0.728 | - National coordinating body: Committee on Antimicrobial Resistance Control (Komite Pengendalian Resistensi Antimikroba) with five working groups, including AMS and infection prevention and control (IPC). Financing mechanism: the national government allocated budget for 2020–2024, aiming for AMR control, including AMS implementation.  - National policies on integrated AMS activities for human health have been issued by Ministry of Health (MoH) and Indonesian Food and Drug Authority: the national formulary, guidelines of antibiotic use (updated in 2021), national guideline on AMS implementation in hospitals that was released in 2021, the smart community movement in using drugs, and standards of pharmacy services in hospitals and pharmacies. Nationwide AMS training for healthcare professionals has been conducted, and participants were rewarded with continuous medical education credit points; regular public awareness campaigns have been conducted including the annual events during World Antimicrobial Awareness Week. The country officially enrolled in WHO Global Antimicrobial Resistance and Use Surveillance System (GLASS) in 2019 for AMR and 2020 for antimicrobial use (AMU).  - Main areas for improvement: lacking a policy on AMS implementation in the primary healthcare; limited coordination between AMS implementation and other relevant programs, such as water sanitation and hygiene (WASH) and universal health coverage (UHC); dispensing over-the-counter medicines, including prescription-only antimicrobials is widespread; a nationwide database for collection, reporting, monitoring, and evaluation (M&E) of AMR, AMU and consumption (AMC), and AMS metrics remains unavailable. |
| Nepal  Population: approximately 31 million;  Population density: 180/km^2^  Gross Domestic Product (nominal) per capita: $1,381  Human Development Index: 0.622 | - National coordinating body: Antimicrobial Resistance Multisectoral Coordination Committee, led by the secretary, overseeing aspects related to AMR containment, including surveillance, AMS, and AMU.  - National Costed-National Action Plan encompassing AMR, AMU, and AMS initiatives has recently been endorsed, aiming to integrate and coordinate all relevant stakeholders and activities; established an electronic Logistics Management Information System to monitor distribution, stock, and consumption of medicines at the local level; efforts to detect and recall substandard drugs through risk-based post-marketing surveillance are in place; regular public awareness campaigns on the responsible use of antibiotics is regularly conducted by the National Health Education Information and Communication Center; established a national surveillance program for AMR with support from international partners. The country officially enrolled in WHO GLASS since 2018 for AMR and 2020 for AMU.  - Main areas for improvement: insufficient dedicated funding from the government for AMS initiatives, relying heavily on international support; implementation of regulations on prescription-only sale/dispensing of antibiotics is partially enforced, with antibiotics occasionally being sold without prescriptions; limited accessibility and mandatory implementation of in-service training on antimicrobial prescribing and AMS for healthcare professionals; guidelines for the use of AMU and AMC data to inform national and institutional decision-making are lacking. |
| Thailand  Population: approximately 66 million;  Population density: 132/km^2^  Gross Domestic Product (nominal) per capita: $7,770  Human Development Index: 0.798 | - The national coordination mechanism of AMR in Thailand has been under the Royal Thai Government and WHO Country Cooperation Strategy Program on AMR, and AMS is one of the main strategies.  - Multiple local guidelines have been generated by multiple stakeholders and societies in Thailand; regular public awareness campaigns have been conducted advocating for the responsible and appropriate use of antibiotics, including the annual events during World Antimicrobial Awareness Week and consistent initiatives promoting responsible antibiotic usage among the public; every hospital incorporates WASH with AMS is integrated into the prevention of Multidrug-Resistant Organisms within WASH training. Infection Control Nurses are frequently involved in both AMS and Infection Prevention and Control (IPC), which includes WASH and Hand Hygiene committees; AMR data is collected by the Department of Medical Sciences and AMC data by the International Health Policy Program. The country officially enrolled in WHO GLASS since 2017 for AMR and 2024 for AMU.  - Areas for improvement: policy and implementation currently lack comprehensive integration between multiple stakeholders; funding for AMS implementation is up to each hospital, and most hospitals have no budget allocated for AMS specifically; planning of improving awareness, education and training to all levels of healthcare officers and to public is still limited; workloads of healthcare officers are high, improvement on surveillance, monitoring and evaluation should avoid increasing workloads of healthcare officers. |
| Viet Nam  Population: approximately 100 million;  Population density: 298/km^2^  Gross Domestic Product (nominal) per capita: $4,806  Human Development Index: 0.766 | - The MoH has established national coordination mechanisms for AMR control with sub-committees to provide oversight and coordinate the activities on specific subject areas including antibiotic use. There is no separate AMS sub-committee under this structure. The coordination of AMS programs is assigned to key staff of the Medical Services Administration within MoH to collaborate with other departments.  - Guidelines have been developed to support healthcare facilities to implement AMS programs including antibiotic treatment guidelines; the new “National Strategy on the Prevention of Antimicrobial Resistance for the period 2023-2030, with a vision to 2045” was recently released, signed by the prime minister; annual nation-wide awareness raising campaigns and related activities organized within and around World Antimicrobial Awareness Week; established a national surveillance program for AMR with support from international partners. The country officially enrolled in WHO GLASS since 2021 for AMR and 2022 for AMU.  - Areas for improvement: lacking funding allocated for the national coordination unit and coordination mechanisms at all levels; weak linkage and integration of AMS principles in existing interconnected programs including WASH, TB, HIV, malaria, universal health care and primary healthcare; national antibiotic use guidelines need to be updated (last update by 2015); no guidelines to support AMS activities in community settings; no system in place to periodically identify the availability of affordable antibiotics at healthcare facilities; dispensing over-the-counter prescription-only medicines still exists, and greater regulatory measures are needed to reduce inappropriate antibiotic use in community settings; WHO AWaRe for antibiotic classification and selection framework has not been applied. |

*^1^Source for country statistics: Wikipedia; ^2^summarized from the national assessment reports*

**Supplemental Table S2. Details on the level of AMS implementation (*number of assessment items* that have been *partially or fully implemented* under each domain) by country based on collected national assessment data, 2022-2023**

| **Domain in each pillar** | | **Detail of implementation status in each pillar** |
| --- | --- | --- |
| **Pillar 1: Establish and develop national coordination mechanisms for antimicrobial stewardship and develop guidelines** | | |
| 1 | Establish and maintain a national coordinating mechanism for AMS that is functional at national, subnational and district levels | AMS-related policies, such as AMR control policy, NAP on AMR, and antibiotic guidelines, were available in four countries. All four countries had formed a national AMR coordination committee, involving stakeholders from government and non-government sectors. Budget allocation for AMR and AMS related activities was included in the NAP (2020-2024) for AMR using a One Health in Indonesia. In Nepal, the NAP being recently endorsed for 2024-2028, with a costed action plan. In Viet Nam, the new strategy on AMR control for 2023-2030 indicates funding for implementation to primarily come from the government budget, and the need to mobilize external funding sources, although the detailed costing plan is not yet publicly available. National antibiotic use guidelines were available in all countries but not updated for Viet Nam since 2015. All four countries lacked a comprehensive approach to address AMR, with limited AMS policy and guidelines for primary healthcare, community settings and private sectors. In Indonesia, a country with a decentralized governance system there was no AMR policy at provincial and district levels. |
| 2 | Develop national treatment and stewardship guidelines, standards and implementation tools |  |
| **Pillar 2: Ensure access to and regulation of antimicrobials** | | |
| 3 | Improve access to essential, quality-assured, safe, effective and affordable antimicrobials | Guidance for monitoring the quality of antimicrobials throughout the supply chain was available in four countries. A prescription-only dispensing regulation for antibiotics in the community was available in all four countries, but with weak enforcement in Indonesia, Nepal and Viet Nam. In these three countries, there was no centralized monitoring system to identify the availability of affordable antibiotics in healthcare facilities, nor a mechanism to report shortages and antibiotic stock-outs. All four countries had adopted the WHO Model List of Essential Medicine. Except for Viet Nam, all other countries had adopted the WHO Access, Watch and Reserve system in the national antibiotic guidelines. |
| 4 | Regulate social triggers and remuneration policies that promote responsible antimicrobial prescription and dispensing behaviours |  |
| 5 | Legislate and regulate responsible and appropriate use and disposal of antimicrobials |  |
| **Pillar 3: Improve awareness, education and training** | | |
| 6 | Improve awareness and engagement to support behavioural change of antimicrobials use | Public awareness campaigns and the World AMR Awareness Week were consistently conducted in four countries. However, impact evaluation of these activities was limited or lacking. AMS principles were integrated into broader education and training pre-service programs for healthcare professionals to some degrees in Nepal, Thailand and Viet Nam; but not yet in Indonesia. |
| 7 | Strengthen health worker capacity through the provision of tailored education and training packages according to health worker roles and functions |  |
| **Pillar 4: Strengthen water, sanitation and hygiene and infection prevention and control** | | |
| 8 | Enhance WASH in health facilities and communities | WASH and IPC programs were implemented to varying levels in four countries^1^. There was representation of AMS staff on the WASH/ IPC programs and/or vice versa in hospital following the national guidelines, but the level of integration also varies across the hospitals and countries. |
| 9 | Implement IPC core components in health facilities |  |
| **Pillar 5: Surveillance, monitoring and evaluation** | | |
| 10 | Surveillance of antimicrobial use and consumption | Surveillance of AMR, AMU and AMC were performed in four countries, in a selection of hospitals and/or laboratories. Four countries have also enrolled in WHO GLASS for both AMR and AMC. Guidelines for the use of AMR, AMU, and AMC data to inform decision-making at the national and institutional levels were only available in Thailand. National frameworks/ indicators for monitoring and evaluation of AMS programs in healthcare facilities were available in four countries. Accountability and feedback mechanisms for AMS programs at the healthcare facilities were established only in Thailand, and to a limited extent in Nepal. |
| 11 | Surveillance of AMR |  |
| 12 | Monitoring and evaluation of AMS activities |  |

*Notes: AMR, Antimicrobial resistance; AMS, Antimicrobial stewardship; AMC, Antimicrobial consumption (usually aggregated data); AMU, Antimicrobial use (usually patient-level data); GLASS, Global Antimicrobial Resistance and Use Surveillance System; ID, Indonesia; IPC, Infection prevention and control; NAP, National Action Plan; NP, Nepal; WASH, Water, sanitation and hygiene; TL, Thailand; VN, Viet Nam; WHO, World Health Organization. ^1^Summary was made based on the participants' responses to the overall questions on WASH and IPC in these two domains of the WHO AMS national assessment tool, not necessarily reflect the detailed assessment of the implementation of the WASH or IPC programs following comprehensive assessments such as WHO's The Infection Prevention and Control Assessment Framework (IPCAF) and the Water and Sanitation for Health Facility Improvement Tool (WASH FIT).*

**Supplemental Table S3. Number and proportion of prescribers and non-prescribers agreeing (combining both “agree” and “strongly agree”) with each of the KAP statement (excluding those with missing data under each KAP statement item) in the study (2022-2023)**

| **KAP statement** | **Prescribers** | **Non-prescribers** |
| --- | --- | --- |
| Optimizing antibiotic use is a priority at my healthcare facility | 144/161 (89%) | 116/132 (88%) |
| Antibiotic use is discussed at facility-wide multidisciplinary team meetings | 130/161 (81%) | 116/132 (88%) |
| I am familiar with the term antibiotic stewardship | 145/161 (90%) | 116/132 (88%) |
| The importance of antibiotic stewardship is communicated (e.g., via posters, emails) at my healthcare facility | 127/161 (79%) | 108/132 (82%) |
| I trust the microbiology test results that I receive at my healthcare facility | 136/161 (84%) | 122/131 (93%) |
| My healthcare facility promptly alerts prescribers about relevant positive culture results (e.g., organism identified in blood culture) to modify antibiotic therapy^1^ | 128/161 (79%) | 118/132 (89%) |
| I am able to access my healthcare facility's updated antibiogram | 97/161 (60%) | **93/132 (70%)** |
| More guidance from the antibiotic stewardship team could improve antibiotic use at my healthcare facility | 147/161 (91%) | 115/132 (87%) |
| Use of broad-spectrum antibiotics when equally effective narrower spectrum antibiotics are available increases antibiotic resistance | 140/161 (87%) | 92/132 (70%) |
| Inappropriate antibiotic use can harm patients | 156/161 (97%) | 125/130 (96%) |
| The incidence of antibiotic-resistant organisms can be reduced by optimizing antibiotic prescribing patterns and infection prevention and control practices | 158/160 (99%) | 128/132 (97%) |
| Appropriate use of antibiotics may reduce antibiotic resistance | 158/160 (99%) | 131/132 (99%) |
| Requiring clinicians to obtain approval prior to prescribing certain antibiotics is a way to improve antibiotic use | 135/160 (84%) | 127/132 (96%) |
| Antibiotics are overused (e.g., antibiotics are used when not clinically indicated) at my healthcare facility | 73/160 (46%) | **44/132 (33%)** |
| Antibiotic resistance is a problem at my healthcare facility | 109/159 (69%) | **87/132 (66%)** |
| There is multidisciplinary teamwork for antibiotic decision-making activities at my healthcare facility | 94/161 (58%) | **93/132 (70%)** |
| I value recommendations from the antibiotic stewardship team at my healthcare facility | 135/161 (84%) | 109/132 (83%) |
| I have access to locally endorsed infectious diseases treatment guidelines used at my healthcare facility | 108/161 (67%) | **89/132 (67%)** |
| I have adequate access to infectious diseases expertise (e.g., infectious diseases trained physician or clinician with experience practicing infectious diseases) at my healthcare facility | 113/161 (70%) | 96/132 (73%) |
| I feel comfortable recommending an intervention to my colleagues on antibiotic use | 109/161 (68%) | **83/132 (63%)** |
| Healthcare workers (e.g., physicians, pharmacists, or nurses) educate patients and/or their families on the use of antibiotics at discharge at my healthcare facility | 130/161 (81%) | 111/131 (85%) |
| I have timely access to microbiology test results and diagnostic information to guide my use of antibiotics | 118/161 (73%) | - |
| Receiving more education on appropriate selection of antibiotic agent, duration of therapy, and dose could improve my antibiotic prescribing practices at my healthcare facility | 152/161 (94%) | - |
| I receive education on how to select the most appropriate antibiotic for treatment based on microbiology test results at my healthcare facility | 107/161 (66%) | - |
| The antibiotic stewardship team can impact my decisions on antibiotic initiation and continuation at my healthcare facility | 121/161 (75%) | - |
| I am pressured to prescribe antibiotics by patients or their families | 27/160 (17%) | - |
| I am pressured to prescribe antibiotics by my colleagues | 20/161 (12%) | - |
| Scientific literature (e.g., published research) influences my decisions on antibiotic prescribing at my healthcare facility | 137/161 (85%) | - |
| Pharmaceutical companies influence some of my decisions on antibiotic prescribing at my healthcare facility | 27/161 (17%) | - |
| I use locally endorsed infectious diseases treatment guidelines when I am making decisions about antibiotic prescribing at my healthcare facility | 129/161 (80%) | - |
| I prescribe certain empiric antibiotics based on consultation with a clinician with experience practicing infectious diseases, infectious diseases trained physician, or the antibiotic stewardship team at my healthcare facility | 136/161 (84%) | - |
| I routinely obtain cultures before starting antibiotic therapy in patients with suspected infection at my healthcare facility | 100/161 (62%) | - |
| I modify my patient's antibiotic treatment after receiving culture and antibiotic susceptibility results when appropriate | 146/161 (91%) | - |
| I consider adverse events when selecting an antibiotic regimen for patients at my healthcare facility | 148/161 (92%) | - |
| I consider drug interactions when selecting an antibiotic regimen for a defined patient population at my healthcare facility | 150/161 (93%) | - |
| I consider my patient's kidney function when dosing antibiotics at my healthcare facility | 157/161 (98%) | - |
| I consider the risk of development of antibiotic resistance in my patients when I prescribe antibiotics | 145/161 (90%) | - |
| I consider the opinion of non-physician staff (e.g., nursing, pharmacy) in antibiotic decision-making at my healthcare facility | 79/161 (49%) | - |
| Receiving feedback about appropriateness of antibiotics that I prescribe could improve my antibiotic prescribing practices | 150/161 (93%) | - |
| Receiving feedback on how my antibiotic prescribing practices compares to my peers could improve my antibiotic prescribing practices | 137/160 (86%) | - |
| I am aware of changes that are needed to my current antibiotic prescribing practices based on feedback received at my healthcare facility | 143/161 (89%) | - |

*Note: Prescribers responded to all 41 statements, non-prescribers responded to the first 21 statements only.*

**Supplemental Figure S2. Correlation between domains within the G-ASET data collected during 2022-2023 from 16 hospitals.** Positive correlations are displayed in a blue scale, a correlation coefficient of one means a perfect positive relationship between two domains, while a coefficient close to zero suggests no significant correlation (negative correlations are displayed in a red scale but no negative correlations were observed in this analysis).

**Supplemental Figure S3. Status of implementation for selected G-ASET items by hospital and country (2022-2023).** For detailed content of each presented item, please refer to the G-ASET form and instructions which is available at US CDC website15. Score 0 = not implemented, 2.5 = partially implemented, 5 = fully implemented; Below is a brief summary of the presented items: 32. at least 4 guidelines were available; 34. guidelines reviewed and updated periodically based on availability of new evidence, with changes communicated to prescribers; 35. AMS team review the facility antibiogram on a regular basis to modify treatment guidelines; 36. at least 9 activities are implemented; 38. standard operating procedures (SOP) for some specific AMS activities such as audit and feedback, guideline development, testing protocols; 39. AMS activity report produced with relevant information; 41. approved antibiotics for use based on the national formulary; 42. lists of restricted antibiotics that require approval by a designated team or person; 45. involved in at least 2 activities; 46. policy that requires prescribers to document antibiotic dose, duration, and indication in the medical record; 47. to facilitate early antibiotic adjustments; 48. technology to identify the most relevant resistance mechanisms (e.g., extended spectrum beta-lactamases, carbapenemases); 49. microbiology provides culture and antibiotic susceptibility testing (AST) results to prescribers in a timely manner (e.g., within 72 hours); 50. utilize selective or cascading antibiotic susceptibility testing reporting (e.g., not reporting an antibiotic that would not be appropriate for the source, not reporting a broad-spectrum antibiotic when a narrower spectrum is available); 51.Microbiology puts comments in culture results to improve antibiotic prescribing; 52. AMS team communicates the emergence of new resistance mechanisms to prescribers; 53. AMS team conducted an analysis of the barriers, challenges and opportunities for AMS implementation.
