# Supplementary material for: Factors influencing antimicrobial stewardship implementation in healthcare facilities in four countries of Southeast Asia: a mixed-methods study (2022–23)
Source: Lancet Reg Health Southeast Asia. 2026 Aug 6;53:100837. doi: 10.1016/j.lansea.2026.100837 (PMC13546922; doi:10.1016/j.lansea.2026.100837)
Supplement: Supplementary file 2 [file mmc2.docx]

***Disclaimer***

***This translation in Vietnamese was submitted by the authors and we reproduce it as supplied. It has not been peer reviewed. Our editorial processes have only been applied to the original abstract in English, which should serve as reference for this manuscript.***

*Tóm tắt*

*Bối cảnh*

Việc triển khai chương trình quản lý sử dụng kháng sinh (QLSDKS) phụ thuộc rất lớn vào bối cảnh cụ thể. Nghiên cứu hỗn hợp này nhằm đánh giá các yếu tố bối cảnh liên quan đến việc triển khai QLSDKS ở cấp quốc gia, cơ sở y tế và nhân viên y tế tại bốn quốc gia châu Á.

*Phương pháp*

Chúng tôi sử dụng công cụ đánh giá QLSDKS cấp quốc gia của Tổ chức Y tế Thế giới tại Indonesia, Nepal, Thái Lan và Việt Nam. Việc triển khai ở cấp cơ sở y tế được đánh giá bằng Công cụ Đánh giá Quản lý Sử dụng Kháng sinh Toàn cầu tại các Cơ sở Y tế Nội trú (G-ASET) của Trung tâm Kiểm soát và Phòng ngừa Dịch bệnh Hoa Kỳ tại 16 bệnh viện thuộc các quốc gia này. Thu thập dữ liệu bao gồm khảo sát cán bộ y tế, phỏng vấn và thảo luận nhóm tập trung.

*Kết quả*

Cả bốn quốc gia đều có chính sách về QLSDKS ở cấp độ quốc gia, trong đó có hai quốc gia đã đưa các cơ chế tài chính vào kế hoạch hành động quốc gia. Các lĩnh vực cần tiếp tục cải thiện gồm xây dựng hướng dẫn QLSDKS cho tuyến chăm sóc ban đầu và cộng đồng; tăng cường liên kết giữa các lĩnh vực và chương trình liên quan; mở rộng đào tạo chuyên môn; củng cố thực thi các quy định; theo dõi hiệu quả một cách hệ thống; và sử dụng dữ liệu tốt hơn cho phản hồi và ra quyết định. Trong 16 bệnh viện, điểm G-ASET trung bình là 241/335 (72%), cao nhất ở lĩnh vực theo dõi, giám sát và báo cáo [56/75 (75%)] và thấp nhất ở đào tạo và giáo dục [17/25 (66%)], và điểm số khác nhau theo loại hình bệnh viện và quy mô giường bệnh. Mặc dù tất cả các bệnh viện đều đã triển khai một số hoạt động QLSDKS, việc thực hiện thường chỉ ở mức một phần, với các khoảng trống trong hướng dẫn điều trị dựa trên dữ liệu, đào tạo nhân viên và nguồn lực.

*Diễn giải*

Các hướng dẫn điều trị và QLSDKS ở cấp quốc gia, cùng với đào tạo nguồn nhân lực, là yếu tố then chốt để tăng cường triển khai chương trình ở cấp cơ sở y tế. Tăng cường phối hợp giữa các chương trình và hỗ trợ cho các bệnh viện quy mô nhỏ cũng như các cơ sở chăm sóc ban đầu ở châu Á sẽ rất cần thiết để thúc đẩy triển khai QLSDKS.

*Tài trợ*

Được hỗ trợ bởi Trung tâm Kiểm soát và Phòng ngừa Dịch bệnh Hoa Kỳ.
